# Bioactive Properties of Composites Based on Silicate Glasses and Different Silver and Gold Structures

**DOI:** 10.3390/ma15051655

**Published:** 2022-02-23

**Authors:** Zsejke-Réka Tóth, János Kiss, Milica Todea, Gábor Kovács, Tamás Gyulavári, Alina Sesarman, Giorgiana Negrea, Dan C. Vodnar, Anna Szabó, Lucian Baia, Klára Magyari

**Affiliations:** 1 Nanostructured Materials and Bio-Nano-Interfaces Center, Interdisciplinary Research Institute on Bio-Nano-Sciences, Babes-Bolyai University, 400271 Cluj-Napoca, Romania; zsejke.toth@ubbcluj.ro (Z.-R.T.); milica.todea@phys.ubbcluj.ro (M.T.); 2 Department of Applied and Environmental Chemistry, University of Szeged, 6720 Szeged, Hungary; kiss.janos505@gmail.com (J.K.); k.gabor84@chem.u-szeged.hu (G.K.); gyulavarit@chem.u-szeged.hu (T.G.); szabo.anna@chem.u-szeged.hu (A.S.); 3 Faculty of Medicine, Iuliu Hațieganu University of Medicine and Pharmacy, 400012 Cluj-Napoca, Romania; 4 Department of Horticulture, Faculty of Technical and Human Sciences, Sapientia Hungarian University of Transylvania, 530104 Târgu-Mureș, Romania; 5 Department of Molecular Biology and Biotechnology, Center of Systems Biology, Biodiversity and Bioresources, Faculty of Biology and Geology, Babes-Bolyai University, 400006 Cluj-Napoca, Romania; alina.sesarman@ubbcluj.ro; 6 Doctoral School in Integrative Biology, Faculty of Biology and Geology, Babes-Bolyai University, 400006 Cluj-Napoca, Romania; giorgiana.negrea@ubbcluj.ro; 7 Faculty of Food Science and Technology, University of Agricultural Science and Veterinary Medicine, 400372 Cluj-Napoca, Romania; dan.vodnar@usamvcluj.ro; 8 Faculty of Physics, Babes-Bolyai University, 400084 Cluj-Napoca, Romania; 9 Institute for Research-Development-Innovation in Applied Natural Sciences, Babes-Bolyai University, 400294 Cluj-Napoca, Romania

**Keywords:** silver, gold nanocages, in vitro bioactivity, antibacterial activity

## Abstract

Using an ideal biomaterial to treat injured bones can accelerate the healing process and simultaneously exhibit antibacterial properties; thus protecting the patient from bacterial infections. Therefore, the aim of this work was to synthesize composites containing silicate-based bioactive glasses and different types of noble metal structures (i.e., AgI pyramids, AgIAu composites, Au nanocages, Au nanocages with added AgI). Bioactive glass was used as an osteoconductive bone substitute and Ag was used for its antibacterial character, while Au was included to accelerate the formation of new bone. To investigate the synergistic effects in these composites, two syntheses were carried out in two ways: AgIAu composites were added in either one step or AgI pyramids and Au nanocages were added separately. All composites showed good in vitro bioactivity. Transformation of AgI in bioactive glasses into Ag nanoparticles and other silver species resulted in good antibacterial behavior. It was observed that the Ag nanoparticles remained in the Au nanocages, which was also beneficial in terms of antibacterial properties. The presence of Au nanoparticles contributed to the composites achieving high cell viability. The most outstanding result was obtained by the consecutive addition of noble metals into the bioactive glasses, resulting in both a high antibacterial effect and good cell viability.

## 1. Introduction

The widespread application of bioactive glasses is not surprising since they are used to treat bone injuries, cancer metastases, and wounds [1]. Moreover, it is known that additional properties can be conferred to bioactive glasses by adding different metals to them [2,3,4]. Among these properties the following ones are worth highlighting: stimulation of angiogenic factors and anti-inflammatory effects, enhancement of cell viability characters, and an increase in antibacterial and osteogenic cellular activity [1,3,5].

The unique properties of gold nanoparticles are related to their (localized) surface plasmon resonance, large surface-to-volume ratio, biocompatibility, low toxicity, and stability [6]. These properties are utilized in several (biomedical) applications [7], such as drug delivery, diagnostics and therapeutics, bionanosensors, and biomedical nanodevices. It is known that gold nanoparticles with an appropriate size and morphology can simulate the proliferation of keratinocytes [8]. Liang et al. [9] reported that gold nanoparticles (with a diameter of 15 nm) loaded into mesoporous on silica nanoparticles promote the viability and proliferation of osteoblasts. Our research group successfully introduced gold nanoparticles into a bioactive glass matrix system [10] without losing their properties. In vivo assays confirmed the following results: (1) the gold nanoparticles containing bioactive glass embedded in Vaseline ointment showed faster wound regeneration in laboratory rats [11]; (2) the gold nanoparticles containing bioactive glass introduced in an alginate–pullulan composite exhibited excellent biocompatibility after in vivo subcutaneous analysis [12].

Unfortunately, surgeries sometime end with a bacterial infection that is received during hospital treatment [13]. Therefore, it is important to develop materials that exhibit antibacterial properties by involving noble metals in them, such as Ag, Pt, Pd, Cu, and Ru [12,14], or metals such as Ti, Co, Zn, and Ni [15,16]. Bioactive glass with Ag nanoparticles (AgNPs) showed high potential in antibacterial applications, as claimed by Bellantone and Hench [17]. After this finding, several studies [18,19,20,21] showed that adding different species of silver into bioactive glass matrices gave the composites antibacterial properties. It has also been stated that AgNPs are one of the best-known noble metals that possess this property but, due to their instability [22], their use still raises a lot of questions. The instability was demonstrated by Leward et al. [23] who shown that silver species in NaCl media transformed into AgNPs and AgCl, which could affect their in vivo performance.

In our previous work [24], we involved AgI particles in a bioactive glass matrix to avoid the possible formation of AgCl after its immersion in simulated body fluid (SBF). In this work, we concluded that AgI transformed into AgNPs, Ca(IO_3_)_2_, and CaI_2_ but still showed acceptable antibacterial properties.

Gold nanoparticles with silver content can be obtained by synthetizing spherical gold nanocages (AuNCs), as small amounts of AgNPs remain in samples that have been used for synthesis [25,26]. By adding the latter to bioactive glass, a moderate antibacterial effect can be obtained [27].

The aim of this work was to prepare biomaterials with bactericidal effects and to maintain the beneficial properties of gold nanoparticles proven in our previous in vivo studies [11,12]. Accordingly, AuNCs and AgI microcrystals were added to bioactive glasses to obtain composites via the sol–gel the method used in our previous studies. Gold and silver particles were added into the bioactive glass using four different pathways: synthesizing two different reference materials, that is, AuNCs and AgI were added separately into the bioactive glass, or two composites were synthesized containing both noble metals. Our investigation on the human epidermal keratinocyte cell (HaCaT) line and the *Pseudomonas aeruginosa* and *Staphylococcus aureus* bacterial strain lines proved that multifunctional bioactive glasses can be successfully synthesized.

## 2. Materials and Methods

### 2.1. Materials 

All chemicals were used as received without further purification. For the synthesis of AgI microcrystals and gold nanocages, ethylene glycol (EG, analytical reagent, Molar Chemical, Halasztelek, Hungary), sodium iodide (NaI, ≥99%, reagent grade, Honeywell, Fluka, Denmark), silver nitrate (AgNO_3_, analytical reagent, 99.8%, Penta Industry, Prague, Czech Republic), polyvinylpyrrolidone (PVP, average mol wt. 40,000, Sigma-Aldrich, Schnelldorf, Germany), ethanol (EtOH, absolute, Molar Chemical, Halásztelek, Hungary), gold(III) chloride trihydrate (HAuCl_4_·3 H_2_O, trace metals basis, Sigma-Aldrich, Schnelldorf, Germany), sodium borohydride (NaBH_4_, 96%, Sigma-Aldrich, Schnelldorf, Germany), sodium citrate dihydrate (Na_3_C_6_H_5_O_7_·2H_2_O, 99.0%, Alfa-Aesar, Haverhill, MA, USA), sodium chloride (NaCl, >99.0%, Spectrum-3D, Debrecen, Hungary), and Pluronic^®^ F-127 (Bioreagent, i.e., suitable for cell culture, Sigma-Aldrich, Schnelldorf, Germany) were used. 

The precursors used for the synthesis of glass were tetraethyl orthosilicate (TEOS, ≥99%, Merck, Burlington, MA, USA), triethyl phosphate (TEP, ≥99%, Merck, Burlington, MA, USA), and calcium nitrate tetrahydrate (Ca(NO_3_)_2_·4H_2_O, ≥99%, Lach-Ner, Neratovice, Czech Republic), which were hydrolyzed in the presence of nitric acid (HNO_3_, 65%, Nordic Chemical, Cluj-Napoca, Romania). Ultrapure water and absolute ethanol were used throughout the whole experimental process. 

For the preparation of SBF sodium chloride (described above), sodium bicarbonate (NaHCO_3_, Penta Industry, Prague, Czech Republic), potassium chloride (KCl, 99.5%, Nordic Chemicals, Cluj-Napoca, Romania), dipotassium hydrogen phosphate (K_2_HPO_4_, 99%, Penta Industry, Prague, Czech Republic), magnesium chloride hexahydrate (MgCl_2_6H_2_O, 99%, Merck, Burlington, MA, USA), calcium chloride (CaCl_2_, >97% Penta Industry, Prague, Czech Republic), sodium sulphate (Na_2_SO_4_, 99%, Nordic Chemicals, Cluj-Napoca, Romania), tris(hydroxymethyl)aminomethane (TRIS, 99.8%, Merck, Burlington, MA, USA), and hydrogen chloride (HCl, 37%, Nordic Chemicals, Cluj-Napoca, Romania) were used.

### 2.2. Synthesis of Silver Iodide and Sperical Gold Nanocages

#### 2.2.1. Synthesis of Silver Iodide 

AgI particles were prepared via solvothermal synthesis by the addition of silver nitrate, sodium iodide, and PVP [24]. The solutions were prepared in two separate beakers. In the first beaker, silver nitrate (0.167 M) was dissolved in 20 mL of EG. In the second beaker, sodium iodide (0.077 M) and PVP (0.0036 M; calculated to units of PVP) were dissolved in 100 mL of EG and stirred for 1 h at 60 °C. After combining the contents of the two beakers, the synthesis mixture was stirred for another hour. A yellowish-green precipitate was formed in an instantaneous reaction, indicating the formation of AgI. After stirring, the suspension was crystallized at 160 °C in an autoclave (160 mL) for 2 h. The sample was then purified with 3 × ≈35 mL Milli-Q water and 1 × ≈35 mL EtOH by centrifugation at 4400 rpm for 10 min, then dried at 40 °C for 12 h. 

#### 2.2.2. Synthesis of Spherical Gold Nanocages

The synthesis of spherical AuNCs was based on the galvanic replacement method in which AgNPs were exchanged [26,27]. In the first part of the synthesis, a sol containing AgNPs was prepared. For this purpose, 227 mL of Milli-Q water was mixed with 33 mL (2.31 mM) of sodium citrate solution. After stirring for 30 min, 2.8 mL (83.67 mM) of silver nitrate solution was added to the mixture. After stirring for another 30 min, the AgNPs were reduced with sodium borohydride (5.25 mL, 0.55 M).

The resulting solution containing AgNPs was transferred to a reflux flask, heated to 100 °C under continuous reflux, and held at that temperature for 10 min. Then, 3.15 mL of 25.39 mM HAuCl_4_·3H_2_O solution was added dropwise and the system was kept at 100 °C for 20 min and refluxed continuously. The solution containing AuNPs was cooled to room temperature and ≈18 g of NaCl was added to facilitate the dissolution of the resulting AgCl. Impurities were removed from the final solution by washing three times with 0.5 mM Pluronic^®^ F-127 polymer solution. 

#### 2.2.3. Synthesis of Silver Iodide with Spherical Gold Nanocages 

A composite of silver iodide with spherical gold nanocages (AgIAu) was fabricated using AgI and AuNCs. First, a sol containing AuNCs was prepared and purified by washing three times with water. Second, AgI was prepared by adding 50 mg of AgI to 100 mL of distilled water under ultrasonication for 15 min. Third, the aqueous gold sol was added to the silver-containing suspension that was washed with 3 × ≈35 mL distilled water in a centrifuge at 4400 rpm for 5 min, then dried at 40 °C for 12 h. For the thermal stability analysis, the AgIAu composite was heat treated at 500 °C in air for 3 h.

### 2.3. Synthesis of Bioactive Glasses with Silver and Gold Content

In the sol–gel derived bioactive glass (BG) noble metals (AgI and AuNCs) were introduced in two different ways: (i) with preliminary composite mixing, i.e, with the addition of AgIAu; and (ii) consecutively added to the system. Reactants were added consecutively after 1 h intervals under continuous stirring, as described in our previous study [27,28]. Finally, the solution of colloidal AgI, AuNCs, AgIAu, AgI, and AuNCs was added and stirred for 1 h. The solutions were stored in a closed container at 37 °C until gelation was reached (~48 h). The obtained gels were aged 3 days at 37 °C, after that they were dried at 110 °C for 24 h. Material stabilization was carried out at 500 °C in air for 3 h. Prior to characterization, the samples were milled by hand in a ceramic mortar.

Table 1 contains the abbreviations of the samples and details about the compositions. In the composites, the AuNCs:AgI ratio was 24:76. Glass composites are traditionally calculated in mol%. For comparison purposes, the amount of silver was expressed in at% in each sample, and the information is presented in Table 1.

### 2.4. Characterization of AgIAu Composites 

The crystalline composition of the AgIAu composites was identified by using a Shimadzu X-ray diffractometer (XRD 6000, Kyoto, Japan), operating with CuKα radiation (λ = 1.54 Å) and a Ni filter. The diffraction patterns were recorded in the 2θ range from 10° to 80° with a speed of 2°/min. The specified band for AgI and the typical localized surface plasmon resonance band for Au nanocages were identified with a Jasco-V780 spectrophotometer equipped with an ILV-724 integrating sphere (UV–Vis, Jasco, Wien, Austria) with spectra resolution of 2 mm. The morphology of the samples was determined with a FEI Technai G2 20 X-TWIN transmission electron microscope (TEM, FEI, Hillsboro, OR, USA) and a Hitachi S-4700 Type II scanning electron microscope (SEM, Hitachi, Tokyo, Japan). 

### 2.5. Structural and Morphological Characterization of Bioactive Glass Composites 

Elemental composition of the glass samples was examined using an energy dispersive X-ray spectroscope (EDX, Hitachi, Tokyo, Japan) with Röntec XFlash Detector 3001 SDD system. The morpho-structural properties of the samples were analyzed by XRD and FT-IR spectroscopy. The FT-IR absorption spectra were recorded with a Jasco 6600 FT-IR spectrometer (IR, Jasco, Tokyo, Japan) within the range of 400–4000 cm^−1^ and a spectra resolution of 4 cm^−1^ by using the KBr pellet technique. 

### 2.6. In Vitro Bioactivity Assays

The in vitro bioactivity assays were analyzed by immersion in SBF (pH = 7.4; 37 °C) for 7 days. An incubator was used to reach 37 °C and the concentration of bioactive glasses was 10 mg·mL^−1^. After 7 days the samples were filtered, rinsed with ultrapure water, and dried. The formation of a hydroxycarbonate apatite (HCA) layer on the surface of the samples was investigate by XRD, FT-IR, UV–Vis, and SEM measurements. FT-IR spectra, XRD diffractograms, and an SEM micrograph were achieved with the same equipment described above.

To analyze surface changes before and after immersion in SBF, XPS spectra were recorded with a SPECS PHOIBOS 150 MCD system employing a monochromatic Al-K_α_ source (1486.6 eV), a hemispherical analyzer, and a charge neutralization device. Samples were fixed on double-sided carbon tape, and care was taken to ensure that the sample particles covered the tape. Experiments were performed by operating the X-ray source with a power of 200 W, while the pressure in the analysis chamber was in the range of 10^−9^–10^−10^ mbar. The binding energy scale was charge referenced to that of C1s at 284.6 eV. Elemental composition was determined from the survey spectra acquired at a pass energy of 60 eV. High-resolution spectra were obtained using an analyzer pass energy of 20 eV. Analysis of the data was carried out with CasaXPS software.

### 2.7. Cell Viability Assay

Cell viability assessment was carried out on a human epidermal keratinocyte cell line (HaCaT, Cell Line Service, Eppelheim, Germany), via the same method that we described in our previous publications [10,27]. The cells were cultured in Dulbecco’s modified Eagle’s medium (Lonza) supplemented with 2 mM L-glutamine, Pen/Strep 100 U/mL, and 10% FC. Then, they were incubated in a humidified incubator under 5% CO_2_ atmosphere at 37 °C. The cytotoxic effects of different samples were assayed with a WST-1 dye (Merk Millipore, Burlington, MA, USA). The HaCaT cells were seeded in a 96-well plate, at a density of 1 × 10^4^ cells/well. The following day, different amounts of the samples were added to the test wells and cells were placed in the incubator for an additional 24 h. All glass samples were tested at three concentrations (75 μg·mL^−1^, 150 μg·mL^−1^, and 300 μg·mL^−1^). Untreated cells were used as controls. At the end of the incubation period, the medium was removed from all wells, and 100 μL of fresh medium containing 10% WST-1 solution was added to each well. The cells were incubated for another 60 min at 37 °C. Empty wells with medium containing WST-1 reagent were used as blanks. Viability of HaCaT cells after 24 h of treatments was determined by measuring the absorbance at 440 nm with a microplate reader (Fluostar Omega, BMG Labtech, Ortenberg, Germany).

### 2.8. Antibacterial Activity

#### 2.8.1. Microorganism and Culture Conditions

One aerobic gram-negative *Pseudomonas aeruginosa* (ATCC 27853) and one gram-positive *Staphylococcus aureus* (ATCC 49444) bacterial strain were used for antibacterial activity analysis. The tested microorganisms were obtained from Food Biotechnology Laboratory, Life Sciences Institute, University of Agricultural Sciences and Veterinary Medicine Cluj-Napoca, Romania. Both bacteria were cultured on Mueller-Hinton Agar and cultures were stored at 4 °C and subcultured once a month.

#### 2.8.2. Microdilution Method

A modified microdilution technique, based on the regulations of the Clinical Laboratory Standard Institute (CLSI), was used to evaluate antimicrobial activity [29]. Bacterial species were cultured overnight at 37 °C in Mueller-Hinton Broth (MHB). The bacterial cell suspensions were adjusted with a 0.9% NaCl sterile saline solution to a concentration of approximately 3 × 10^5^ colony-forming units (CFU)/mL in a final volume of 100 µL per well. The inoculum was stored at +4 °C for further use. Dilutions of the inoculum were cultured on solid Mueller–Hinton (MH) for bacteria to verify the absence of contamination and check the inoculum’s validity. Determinations of minimum inhibitory concentrations (MICs) were performed by a serial dilution technique using 96-well microtitre plates. The samples were transferred to the wells containing 100 μL MHB, and afterward, 10 μL of inoculum was added to all the wells. The microplates were incubated for 18 h at 37 °C. The MIC of the samples were detected following the addition of 20 μL (0.2 mg·mL^−1^) of resazurin solution to each well, and the plates were incubated for 2 h at 37 °C. A change from blue to pink indicated a reduction in resazurin and, therefore, bacterial growth. The MIC was defined as the lowest drug concentration that prevented this color change. The concentration of the samples was 10 mg·mL^−1^. A control sample gentamicin solution of the same concentration was used for the tested samples. 

### 2.9. Statistical Analysis 

All data in the cell viability assay were reported as the mean ± SD of triplicate measurements. The values were analyzed by two-way analysis of variance ANOVA. Statistical significance was at *p* < 0.05 in all cases. Statistical values were obtained using GraphPad Prism 8.0 software. The antimicrobial analysis required no statistical validation as the method was one of the highest confidence interval methods [30]. 

## 3. Results and Discussion

### 3.1. Characterization of The Composites Containing AgI Microcrystals, Spherical Gold Nanocages, or AgIAu Nanostructures

**Figure 1:**
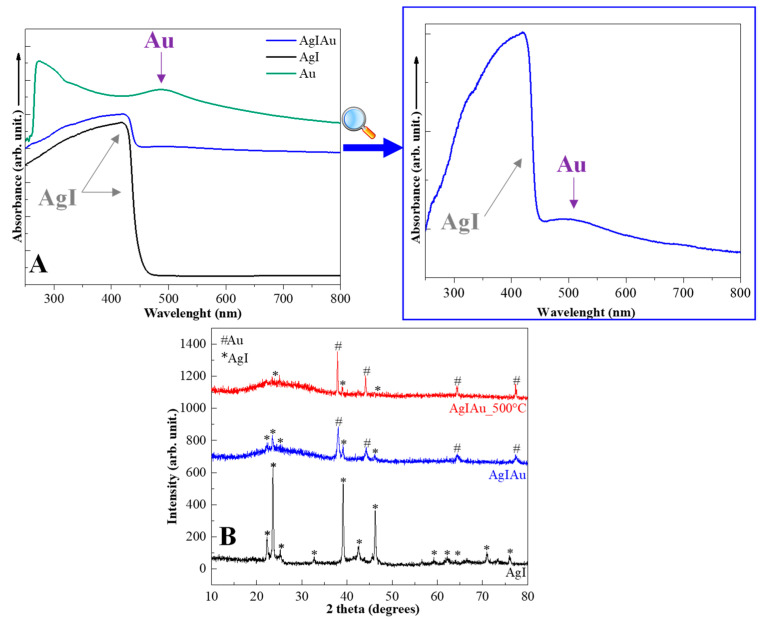
The characterization of the Au nanocages, AgI microcrystals, and AgIAu composite: (**A**) UV–Vis spectra and (**B**) XRD patterns.

**Figure 2:**
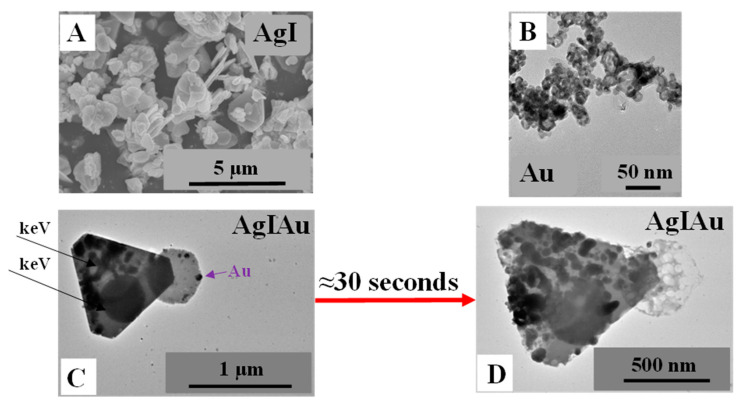
SEM micrograph of pyramidal structure of AgI (**A**); TEM micrographs of Au nanocages (**B**); and AgIAu composites before (**C**) and after electron beam irradiation (**D**).

After the synthesis of AgI microcrystals and AuNCs, their characterization was carried out to investigate their reproducibility. The results were compared with those published in our previous work [24,27]. The UV–Vis spectra, XRD patterns, and SEM micrographs of the AgI microcrystals confirmed the reproducibility of the samples as follows: (i) in the UV–Vis spectrum (Figure 1A) of the sample the typical absorption band for AgI (415–480 nm; [31]) was identified; (ii) in Figure 1B the corresponding reflections of AgI (β-AgI—COD 00-101-1025 and γ-AgI—COD 00-901-1693) were observed; and (iii) the SEM images confirmed the pyramidal structure (Figure 2A). It should be emphasized that AgI was semicrystalline and the two different crystal phases of AgI could not be distinguished because their reflections overlapped with each other [32]. 

The UV–Vis spectra of the AuNCs showed the appropriate (localized) surface plasmon resonance band of AuNCs, located at ≈490 nm (Figure 1A). In the TEM micrographs the presence of Au nanostructures of about 18 ± 3 nm were also observed (Figure 2B). The first proof of successful composite mixing was obtained by UV–Vis measurements. Both the (localized) surface plasmon resonance band of AuNCs (located at 500 nm; insert graph Figure 1A) and the absorption band of AgI (between 350–470 nm) could be observed. The second proof was obtained by XRD measurements, revealing that not only could the AgI reflection be identified but the reflection of Au (COD 00-900-8463) could also be identified. Moreover, in the TEM images small particles could be observed on the surface of the AgI microparticles (Figure 2C). Unfortunately, the low stability of silver-based materials could also be noticed in Figure 2D as the surface of the particles changed with the energy of the electron beam, thereby their size could not be analyzed.

In our previous work [27] it was shown that the gold nanocages introduced into the bioactive glasses were recrystallized into spherical gold nanoparticles by heat treatment at 500 °C. It has also been demonstrated by our group [24] that AgI microcrystals remained stable after heat treatment at 500 °C. Thermal stability of the AgIAu composite was investigated to determine possible transformations during heat treatment; thus, the composite was heat treated at 500 °C. After heat treatment, the reflection for Au and AgI were still presented, the gold became crystalline, and the AgI did not have any significant changes (Figure 1B). 

### 3.2. Characterization and In Vitro Bioactivity of Bioactive Glass Composites

**Figure 3:**
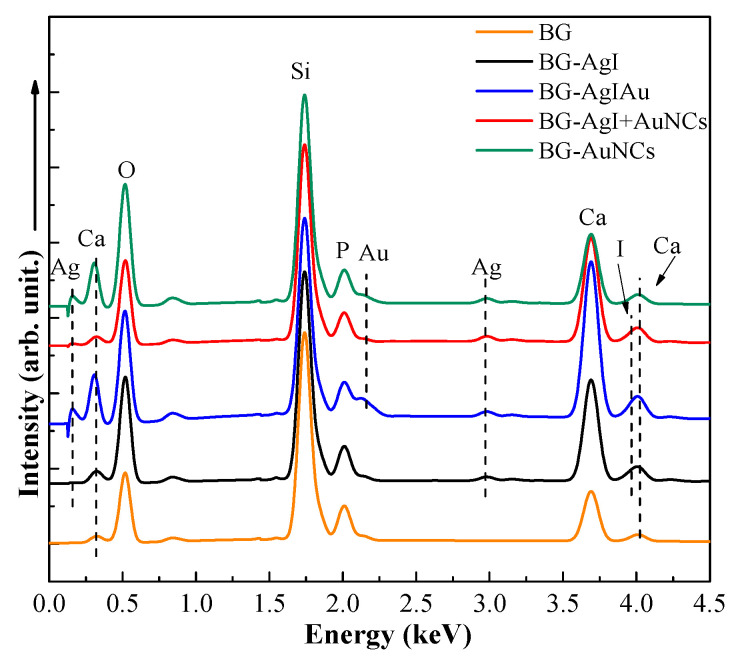
EDX spectra of the BG, BG-AgI, BG-AgIAu, BG-AgI+AuNCs, and BG-AuNCs samples.

EDX analysis was performed for elemental identification of composites. In Figure 3 the presence of all metal components can be seen. The silver and iodine values were comparable with theoretical values (Table 2). The presence of gold was also confirmed, but due to the small amount the obtained value differed to the theoretical one. 

**Figure 4:**
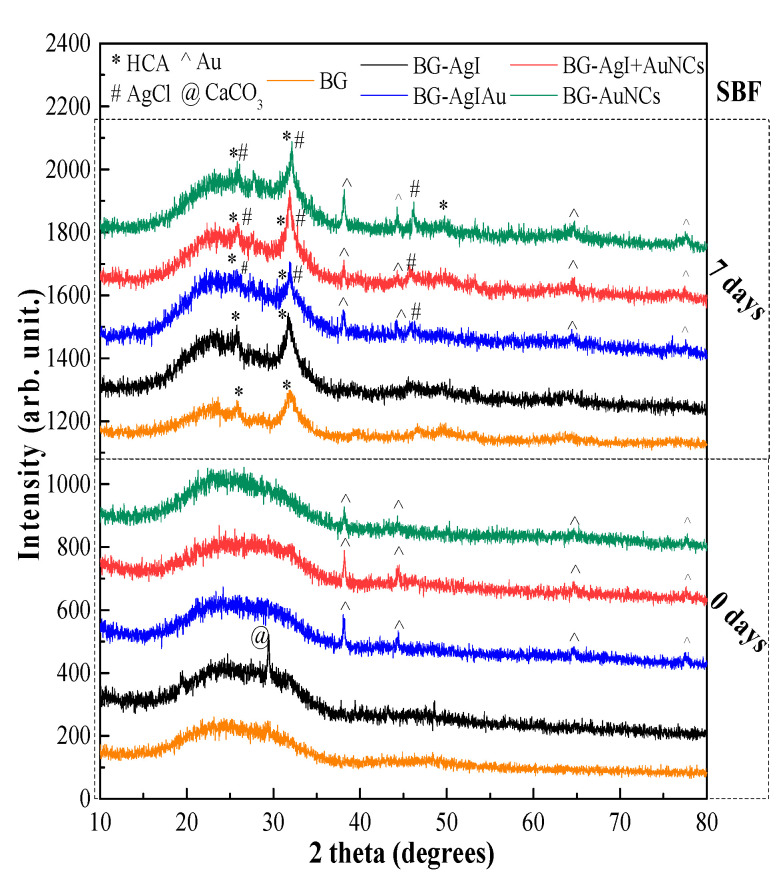
XRD patterns of the BG, BG-AgI, BG-AgIAu, BG-AgI+AuNCs, and BG-AuNCs samples before (0 days) and after (7 days) immersion in SBF.

The XRD patterns (Figure 4) of gold-containing samples exhibited characteristic reflections of metallic gold (COD 00-900-8463). These signals were recorded both before and after immersion in SBF. In the BG-AgI sample an additional reflection was observed at 29.45 (2θ°). This could be attributed to the presence of CaCO_3_ (calcite) (COD 00-101-0928), which only appeared after the heat treatment of the sample. The formation of CaCO_3_ could have originated from the high concentration of calcium ions and the textural parameters of the bioactive glasses [33].

After immersion in SBF for 7 days, all samples showed reflections associated with the presence of an HCA layer precipitated on the surface of the samples (COD file no. 09-0432). After soaking in SBF, AgCl (COD file no. 00-901-1666) was formed on the surface of the silver containing BG composites because of the high amount of NaCl in SBF [34]. The formation of AgCl occurred in the BG–AuNCs samples when the amount of Ag was low. 

**Figure 5:**
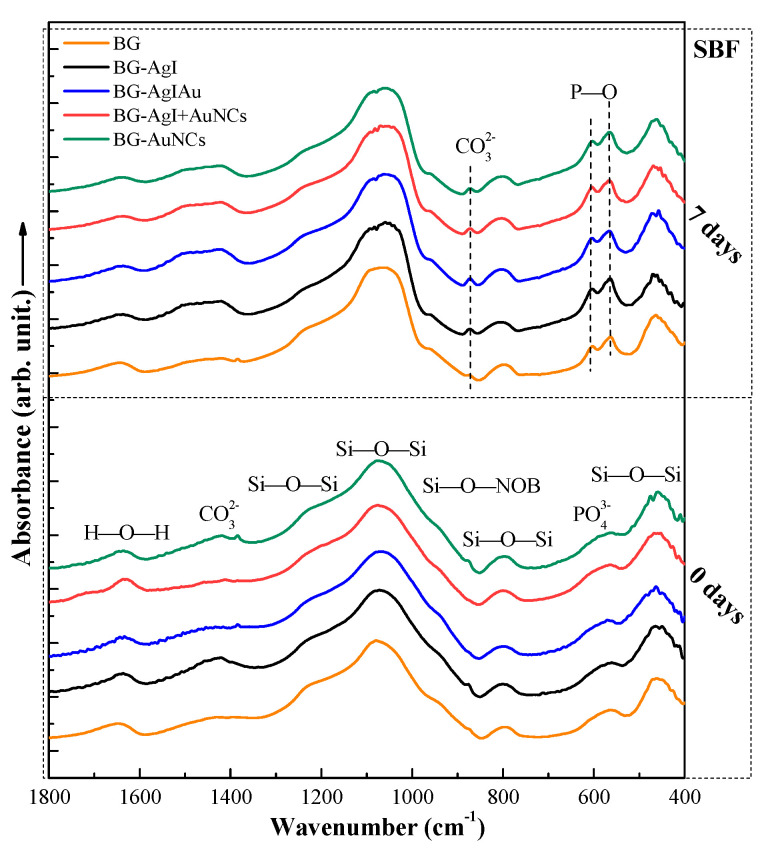
FT-IR spectra of the BG, BG-AgI, BG-AgIAu, BG-AgI+AuNCs, and BG-AuNCs samples before (0 days) and after (7 days) immersion in SBF.

The formation of BG was confirmed (Figure 5) by the presence of an absorption band at 1080 cm^−1^ and a shoulder around 950 cm^−1^. The former could be attributed to the stretching vibration of Si-O bonds, while the latter to the vibrations of Si-O-Si groups [35]. The strong absorption band around 470 cm^−1^ was attributed to the torsional vibrations of the Si-O-Si groups, which also confirmed the presence of BG. The FT-IR spectra of the all samples immersed in SBF for seven days showed a doublet at 604 and 564 cm^−1^. These could be attributed to the vibrations of [PO_4_] units corresponding to crystalline HCA (Figure 5) [36]. In addition to the phosphate bands, a new band at 870 cm^−1^ was observed that could be ascribed to carbonate ions indicating the formation of a carbonate apatite phase.

**Figure 6:**
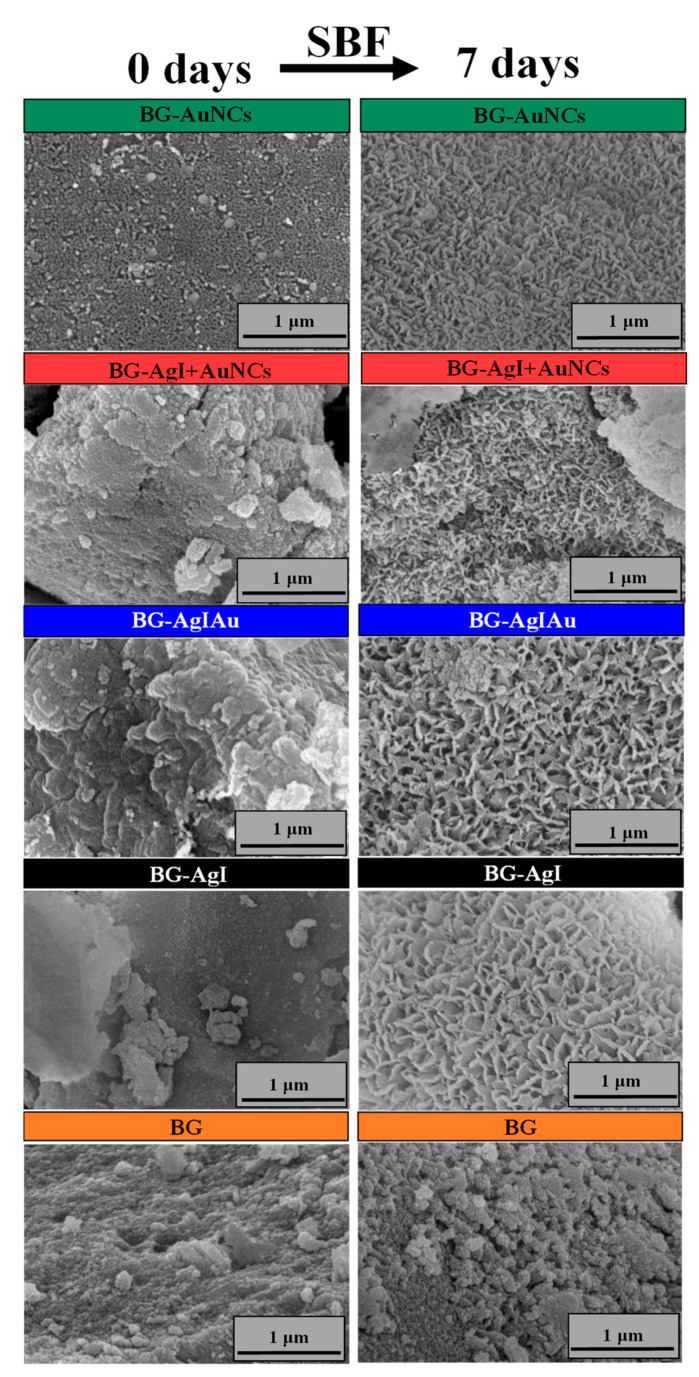
SEM micrographs BG, BG-AgI, BG-AgIAu, BG-AgI+AuNCs, and BG-AuNCs before (0 days) and after (7 days) immersion in SBF.

SEM images (Figure 6) confirmed that the BG had a porous structure before immersion in SBF. It was also ascertained that samples having a cauliflower-like structure showed good bioactivity. These structures confirmed the formation of the HCA layer on the surface of the BG composites.

**Figure 7:**
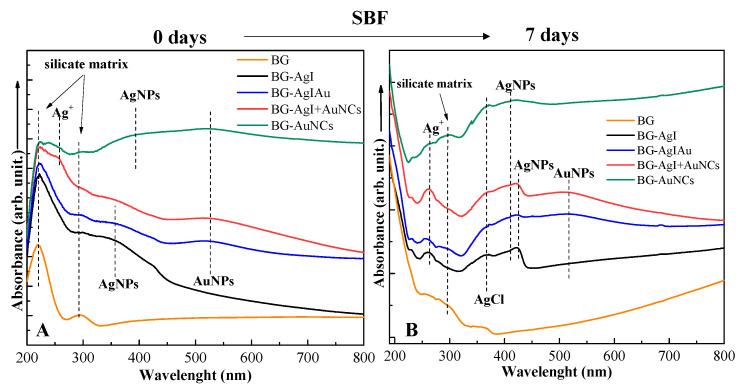
UV–Vis spectra of BG composites: (**A**) before (0 days) and (**B**) after (7 days) immersion in SBF.

The plasmon resonance band of AgNPs (≈357 nm) and gold nanoparticles (≈525 nm) could be identified in the UV–Vis spectra (Figure 7A) of the BG composites before immersion in SBF. The plasmon resonance band of AgNPs (located at ≈394 nm) could also be identified in the BG-AuNCs composites. In these composites, a low amount of remaining silver was observed because their synthesis involves the addition of AgNPs as well. Ag^+^ ions, with a signal around 260 nm, were visible only in the BG-AuNCs and BG-AgI+AuNCs. The similarity of these two samples was due to the fact that in both cases, AuNCs were added into the BG structure. As described before, the Ag^+^ ions could originate from the silver that remained after the synthesis of AuNCs. The presence of Ag^+^ ions could not be observed in the XRD patterns either because their amount was low or they were present in the amorphous phase. UV–Vis absorption bands around 290 and 250 nm of the BG samples’ spectra appeared to be due to the presence of Si-O-(Si, Ca) and P-O-(P, Ca) groups, respectively [37].

After immersion in SBF for 7 days, the surface plasmon resonance of gold nanoparticles (at ≈414 nm and ≈ 425 nm) and the absorption band of Ag^+^ ions (at around 260 nm) were detected (Figure 7B). In addition, a band associated with the presence of AgCl was observed at 369 nm. The surface plasmon resonance signal was observed for the sample containing AuNCs. However, most probably these bands originated from the AgNPs that remained in the system during the galvanic replacement of AuNCs. For the AgI-containing samples, an additional plasmon resonance band correspond to AgNPs could be detected, presumably due to the transformation of AgI.

XPS measurements were also carried out to analyze the chemical state of the samples’ surfaces and to provide the presence of the HCA layer. The proof for the formation of a HCA layer was that the atomic percentage of Si decreased after the immersion into SBF (Table 3). Another proof was the increase in the atomic percentages of Ca and P (Table 3). It is known that XPS measurements can only provide information from the surface of a sample (≈10 nm). Thus, based on the decreasing amount of Si, and the increase in Ca and P amounts, the HCA layer was formed on the surface of the composites [24]. The obtained Ca/P ratio suggested that the surface layer contained carbonates and non-stoichiometric components (decreasing from 2.3 to 1.33) [38]. 

**Figure 8:**
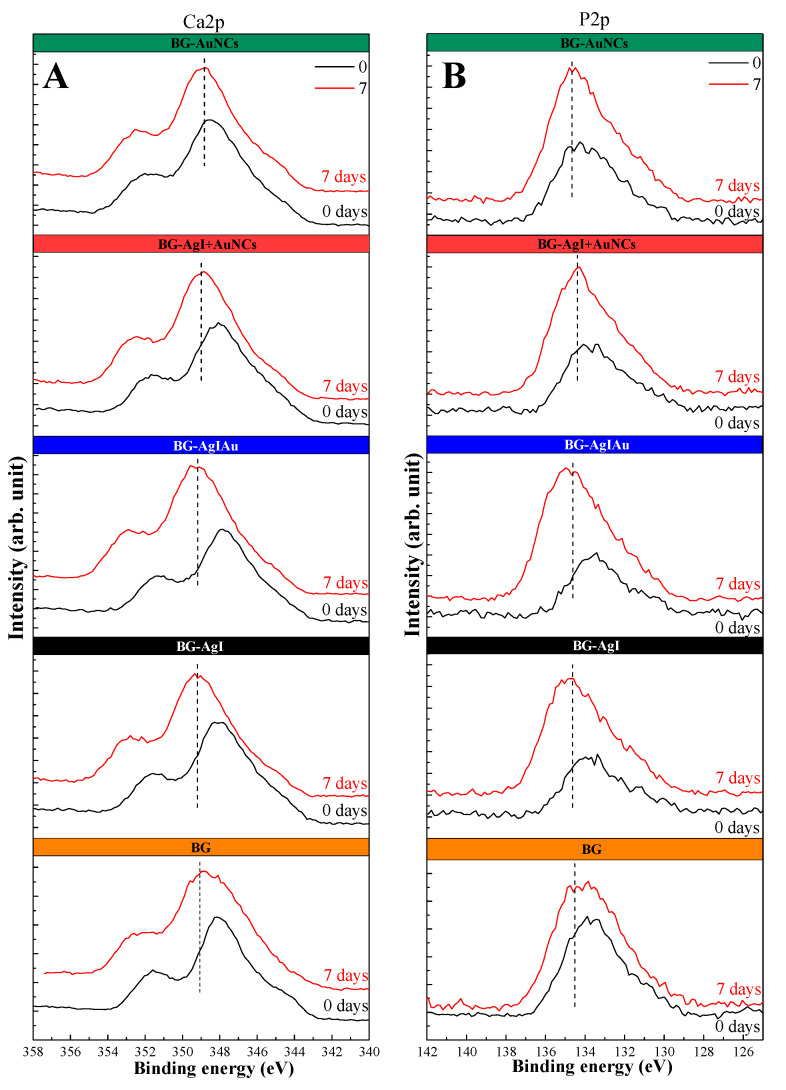
XPS core level spectra for Ca2p (**A**) and P2p (**B**) in BG composites.

High-resolution Ca2p and P2p spectra were also recorded (Figure 8), and the binding energy of 348 eV and 351.5 eV were attributed to Ca2p orbitals, while the one at 133 eV to P2p orbitals [24,38]. After immersion in SBF, both photoelectron peaks broadened and their position changed: for Ca2p, it changed from ≈348 eV to ≈349.3 eV, and from 351.5 eV to 352.64 eV; while for P2p, it changed from ≈133 eV to ≈135 eV. The broadening of the Ca2p peak could be due to the transformation of CaO into CaI_2_ and Ca(IO_3_)_2_ as was proved in our previous study [24]. These transformations were not surprising since the presence of AgNPs was confirmed based on the UV–Vis spectra (Figure 7). This meant that the AgI in the BG transformed into AgNPs and other species of silver and into CaI_2_ and CaIO_3_. Moreover, for the BG-AuNCs the degree of these broadenings was low and could be attributed to the formation of the apatite layer (Figure 8).

### 3.3. Influence of Silver and Gold on Cell Viability and Antibacterial Activity

**Figure 9:**
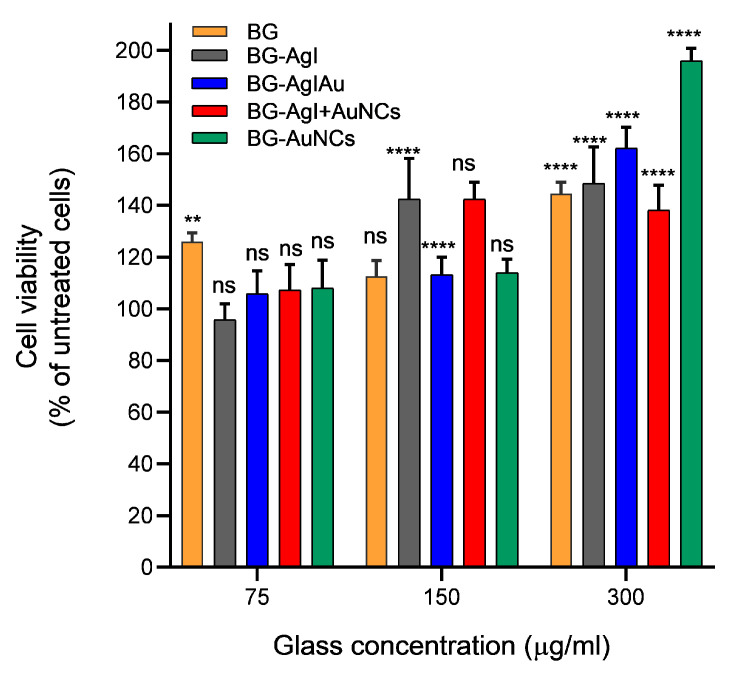
Viability of HaCaT cells in the presence of BG, BG-AgI, BG-AgIAu, BG-AgI+AuNCs, and BG-AuNCs in different concentrations. * *p* < 0.05.

The toxicity of the silver content was evaluated via a cell viability assay following the viability of HaCaT cells in contact with the BG samples for 24 h. First, it needs to be mentioned that, independently of the BG composites used, and the different concentration of glasses, all samples showed cell viability greater than 100% (Figure 9). Therefore, it could be concluded that the composites were not cytotoxic.

**Figure 10:**
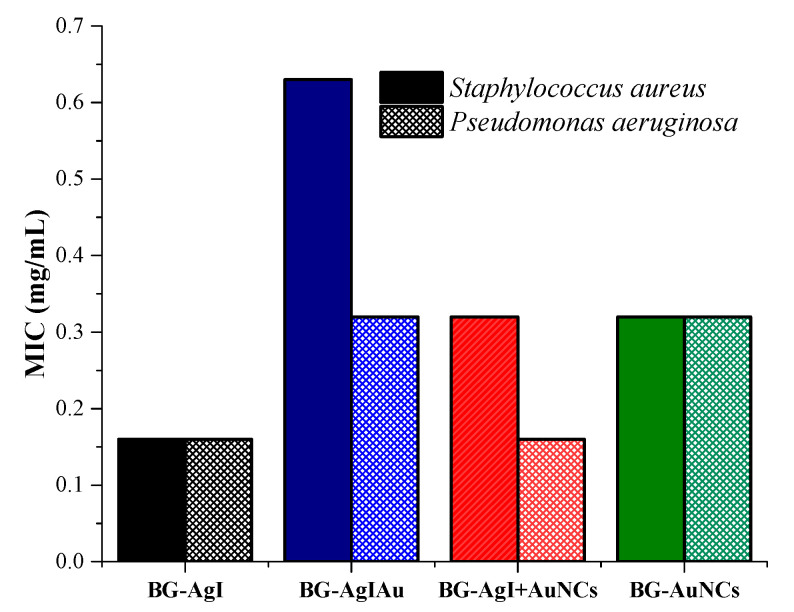
Minimal inhibitory concentration (MIC) of *S. aureus* and *P. aeruginosa* using BG composites. Gentamycin was used as a positive control obtaining MIC of 0.0024 mg∙mL^−1^ for both bacteria.

The next step of our research was to investigate the antibacterial character of the samples using two different bacterial strains: *S. aureus* (gram-positive; Figure 10, self-colored bar) and *P. aeruginosa* (gram-negative; Figure 10, bar with pattern). Since the gram-negative bacterial strains had a thinner cell wall than that of *S. aureus*, the outstanding antibacterial character of the samples was not surprising. BG-AgI showed the highest antibacterial character, which could have originated from the transformation of AgI into AgNPs, as confirmed by the UV–Vis spectra (Figure 7A). Reasonable antibacterial activity was observed for BG-AuNCs, which could be due to the low amount of silver, as confirmed by the UV–Vis spectra (Figure 7A). Composites of BG-AgIAu and BG-AgI+AuNCs showed good antibacterial activity against *P. aeruginosa*. The composites, where the noble metal-based particles were added consecutively (BG-AgI+AuNCs) showed a higher resistance to *S. aureus*, from which it was concluded that this approach was best. The silver-free glass sample has no antibacterial effect (data not shown).

Based on the results, we could conclude that the addition of Ag and Au enabled the bioactive glasses with both antibacterial activity and with the ability to enhance the viability of HaCaT cells. Furthermore, the best combination was obtained when silver and gold content were consecutively added into the BG instead of creating a composite out of them beforehand by mixing.

## 4. Conclusions

Four different composites were prepared to investigate the synergistic effect between bioactive glass composites and two noble metals. From these four samples two were synthesized as reference materials: one containing only silver iodide and the other one containing predominantly Au nanocages. Another two samples were fabricated that contained both noble metals, either by adding them consecutively or as a AgIAu composite. In addition, noble-metal-free bioactive glass was synthetized for comparison purposes. Based on morphological and structural characterizations, bioactive glasses with amorphous structures were successfully obtained independently of the noble metal or noble-metal composites used. All composites presented excellent bioactivity and induced the proliferation of HaCaT cells. Composites with silver content possessed good antibacterial activity against the *S. aureus* and *P. aeruginosa* bacterial strains. The best results were achieved when silver iodide and gold nanocages were added consecutively to the bioactive glasses, resulting in versatile bioactive glasses. 

## Figures and Tables

**Table 1:** Abbreviation and composition of glasses.

Glass Samples (mol%)	Abbreviation	Silver Precursors	Silver Component Amount (mol%)	Silver Component Amount (at%)	Gold Component Amount (at%)
60SiO_2_⋅32CaO·8P_2_O_5_	BG	–	–	–	–
60SiO_2_⋅31.25CaO·8P_2_O_5_·0.75AgI	BG-AgI	Silver iodide	0.75	0.25	–
60SiO_2_⋅31.1CaO·8P_2_O_5_·(0.75AgI+0.15Au_2_O) ^§^	BG-AgI+AuNCs	Silver iodide **and** gold nanocages	0.75	0.249	0.089 *
60SiO_2_⋅31.1CaO·8P_2_O_5_·(0.75AgI-0.15Au_2_O) ^§^	BG-AgIAu	Silver iodide **with** gold nanocages	0.75	0.249	0.09
60SiO_2_⋅31.85CaO·8P_2_O_5_·0.15Au_2_O ^§^	BG-AuNCs	Gold nanocages	–	0.0024	0.089 *

^§^ the amount of gold in the glasses is conventionally indicated as the oxidation state of gold, that is Au_2_O. * the amount of silver introduced during the synthesis of gold nanocages was approximately 0.0024 at%.

**Table 2:** Si, Ag, I, and Au content of the heat-treated BG composites obtained from EDX measurements. For comparison, theoretical values were also calculated.

Sample		Elements (at%)			
		Si	Ag	I	Au
BG	Theoretical	20.00	-	-	-
	Heat-treated	21.76	-	-	-
BG-AgI	Theoretical	20.00	0.25	0.25	-
	Heat-treated	18.51	0.17	0.25	-
BG-AgIAu	Theoretical	20.00	0.24	0.24	0.089
	Heat-treated	14.97	0.17	0.14	0.015
BG-AgI+AuNCs	Theoretical	20.00	0.24	0.24	0.09
	Heat-treated	16.10	0.3	0.22	0.015
BG-AuNCs	Theoretical	20	0.0024	-	0.089
	Heat-treated	17.82	0.22	-	0.074

**Table 3:** Si, Ca, and P content of the heat-treated and immersed BG composites obtained from XPS measurements. For comparison, theoretical values were also calculated.

Sample		Elements (at%)			Ratio
		Si	Ca	P	Ca/P
BG	Theoretical	20.00	10.66	5.33	2
	Heat-treated	21.1	9.6	7.7	1.24
	Immersed in SBF	16.4	11.8	9.4	1.25
BG-AgI	Theoretical	20.00	10.41	5.33	1.95
	Heat-treated	16.30	9.20	4.00	2.30
	Immersed in SBF	8.40	12.80	9.60	1.33
BG-AgIAu	Theoretical	20.00	10.36	5.33	1.94
	Heat-treated	15.67	8.50	2.90	2.93
	Immersed in SBF	2.30	16.30	11.70	1.39
BG-AgI+AuNCs	Theoretical	20.00	10.36	5.33	1.94
	Heat-treated	20.46	10.40	4.40	2.36
	Immersed in SBF	6.9	13.7	10.2	1.34
BG-AuNCs	Theoretical	20	10.61	5.33	1.99
	Heat-treated	15.8	12.8	6.3	2.03
	Immersed in SBF	3.9	15.8	12.1	1.31

## Data Availability

Not applicable.
